# Iraq/Afghanistan war lung injury reflects burn pits exposure

**DOI:** 10.1038/s41598-022-18252-2

**Published:** 2022-08-29

**Authors:** Timothy Olsen, Dennis Caruana, Keely Cheslack-Postava, Austin Szema, Juergen Thieme, Andrew Kiss, Malvika Singh, Gregory Smith, Steven McClain, Timothy Glotch, Michael Esposito, Robert Promisloff, David Ng, Xueyan He, Mikala Egeblad, Richard Kew, Anthony Szema

**Affiliations:** 1 University of Rochester School of Medicine and Dentistry, Simon Business School, University of Rochester, Rochester, USA; 2 Yale University School of Medicine, New Haven, USA; 3 Columbia University Global Psychiatric Epidemiology Group, NYSPI Columbia University Department of Psychiatry, New York, USA; 4 Northeastern University College of Art, Media, and Design (CAMD) Game Design Program, Boston, USA; 5 Brookhaven National Laboratory National Synchrotron Light Source II Beam ID-5, Upton, USA; 6 Science Coordinator Imaging and Microscopy Program and Department of Geosciences, Stony Brook University, Stony Brook, USA; 7 Brookhaven National Laboratory National Synchrotron Radiation Light Source II Bean ID-5, Upton, USA; 8 Department of Pharmacological Sciences, Stony Brook University, Stony Brook, USA; 9 McClain Laboratories, Smithtown, USA; 10 Center for Space Exploration (CEx) Department of Geosciences, Stony Brook University, Stony Brook, USA; 11 Department of Pathology North Shore University Hospital Northwell Health, Donald and Barbara Zucker School of Medicine at Hofstra/Northwell, Hempstead, USA; 12 Drexel University College of Medicine, Philadelphia, USA; 13 Rockefeller University Department of Cancer Biology, New York, USA; 14 Cold Spring Harbor Laboratory Department of Cancer Biology, Cold Spring Harbor, New York, USA; 15 Department of Pathology Stony Brook University, Stony Brook, NY USA; 16 Division of Pulmonary and Critical Care, Division of Allergy/Immunology, Northwell Health, New Hyde Park, USA; 17 Department of Occupational Medicine, Epidemiology and Prevention, International Center of Excellence in Deployment Health and Medical Geosciences, Donald and Barbara Zucker School of Medicine at Hofstra/Northwell, Hempstead, USA

**Keywords:** Physiology, Environmental sciences, Environmental social sciences, Pathogenesis, Risk factors

## Abstract

This descriptive case series retrospectively reviewed medical records from thirty-one previously healthy, war-fighting veterans who self-reported exposure to airborne hazards while serving in Iraq and Afghanistan between 2003 and the present. They all noted new-onset dyspnea, which began during deployment or as a military contractor. Twenty-one subjects underwent non-invasive pulmonary diagnostic testing, including maximum expiratory pressure (MEP) and impulse oscillometry (IOS). In addition, five soldiers received a lung biopsy; tissue results were compared to a previously published sample from a soldier in our Iraq Afghanistan War Lung Injury database and others in our database with similar exposures, including burn pits. We also reviewed civilian control samples (5) from the Stony Brook University database. Military personnel were referred to our International Center of Excellence in Deployment Health and Medical Geosciences, Donald and Barbara Zucker School of Medicine at Hofstra/Northwell under the auspices of Northwell IRB: 17-0140-FIMR Feinstein Institution for Medical Research “Clinicopathologic characteristics of Iraq Afghanistan War Lung Injury.” We retrospectively examined medical records, including exposure data, radiologic imaging, and non-invasive pulmonary function testing (MGC Diagnostic Platinum Elite Plethysmograph) using the American Thoracic Society (ATS) standard interpretation based on Morgan et al*.*, and for a limited cohort, biopsy data. Lung tissue, when available, was examined for carbonaceous particles, polycyclic aromatic hydrocarbons (Raman spectroscopy), metals, titanium connected to iron (Brookhaven National Laboratory, National Synchrotron Light Source II, Beamline 5-ID), oxidized metals, combustion temperature, inflammatory cell accumulation and fibrosis, neutrophil extracellular traps, Sirius red, Prussian Blue, as well as polarizable crystals/particulate matter/dust. Among twenty-one previously healthy, deployable soldiers with non-invasive pulmonary diagnostic tests, post-deployment, all had severely decreased MEP values, averaging 42% predicted. These same patients concurrently demonstrated abnormal airways reactance (X5Hz) and peripheral/distal airways resistance (D5–D20%) via IOS, averaging − 1369% and 23% predicted, respectively. These tests support the concept of airways hyperresponsiveness and distal airways narrowing, respectively. Among the five soldiers biopsied, all had constrictive bronchiolitis or bronchiolitis or severe pulmonary fibrosis. We detected the presence of polycyclic aromatic hydrocarbons (PAH)—which are products of incomplete combustion—in the lung tissue of all five warfighters. All also had detectable titanium and iron in the lungs. Metals were all oxidized, supporting the concept of inhaling burned metals. Combustion temperature was consistent with that of burned petrol rather than higher temperatures noted with cigarettes. All were nonsmokers. Neutrophil extracellular traps were reported in two biopsies. Compared to our prior biopsies in our Middle East deployment database, these histopathologic results are similar, since all database biopsies have constrictive bronchiolitis, one has lung fibrosis with titanium bound to iron in fixed mathematical ratios of 1:7 and demonstrated polarizable crystals. These results, particularly constrictive bronchiolitis and polarizable crystals, support the prior data of King et al. (N. Engl. J. Med. 365:222–230, 2011) Soldiers in this cohort deployed to Iraq and Afghanistan since 2003, with exposure to airborne hazards, including sandstorms, burn pits, and improvised explosive devices, are at high risk for developing chronic clinical respiratory problems, including: (1) reduction in respiratory muscle strength; (2) airways hyperresponsiveness; and (3) distal airway narrowing, which may be associated with histopathologic evidence of lung damage, reflecting inhalation of burned particles from burn pits along with particulate matter/dust. Non-invasive pulmonary diagnostic tests are a predictor of burn pit-induced lung injury.

## Introduction

Since 2003, previously-healthy Iraq and Afghanistan war veterans began to self-report new-onset respiratory symptoms associated with deployment^1–7^. Etiologic mechanisms have been plausibly linked to exposure to airborne hazards, notably: (1) sandstorms; (2) improvised explosive devices (IEDs) with associated “blast overpressure” or shock waves; (3) inhaled metals from explosions; (4) aeroallergens; and (5) burn pits. The latter are sites located on deployment bases used to burn massive amounts of waste in open-air fashion without incinerators^8^. JP-8, jet fuel, is often used as ignition for these burn pits (or garbage fires) despite containing benzene (a carcinogen) that is released along with small particulate matter (PM) from products of incomplete combustion^9,10^. Materials burned included chemicals, paints, medical and human waste, computers/electronics, plastic water bottles, munitions, and vehicles^11,12^. The resulting generated byproducts included: small particulate matter (PM), n-hexane, dioxins, sulfur dioxide, furans, and heavy metals (known byproducts released from the combustion of plastics)^13^, benzene (from JP-8 jet fuel), polycyclic aromatic hydrocarbons (PAH which are products of incomplete combustion and are carcinogenic)^9,14,15^, and burned metals (pro-fibrotic)^11,16,17^. Burn pit emissions/plume inhaled with air-suspended sand and dust can induce lung injury in mice^16,18^.

The Department of Veterans Affairs has denied 78% of disability claims for toxic exposures related to burn pits^19^. “Between 2007 and 2020, VA approved disability claims related to burn-pit exposure for 2828 veterans out of 12,582, according to Laurine Carson, deputy executive director of policy and procedures for the VA^19^.” The National Academy of Sciences indicates that “[veterans’] respiratory symptoms had limited or suggestive evidence of an association with airborne hazards, while the other categories of health outcomes had inadequate or insufficient evidence to determine an association with changes in pulmonary function and noninfectious lower airway and interstitial lung diseases…”^20^ Moreover, the Veterans Affairs website until 2020 states “there is not enough medical or scientific information on potential for long-term health effects in service members caused by exposures to smoke from burn pits.”^21^ While the 2021 US Senate bill, “True Cost of War Recognition Act (TCWR),” aimed to provide presumptive coverage for nine respiratory illnesses, diagnostic proof of several listed conditions warrants sophisticated measures^22^. For example, diagnosis of constrictive bronchiolitis may require a surgical lung biopsy, which may be associated with attendant morbidity and mortality.

Our current study presents an association between exposure to military airborne hazards in Iraq and Afghanistan, linking combustion products from burn pits and particulate matter with chronic pulmonary diseases. We highlight less commonly employed non-invasive and invasive approaches for detecting new-onset respiratory diseases in veterans who were deployed to Iraq and Afghanistan between 2003 and 2021.

## Methods

### Study design

In an anonymously coded, de-identified fashion, we retrospectively examined a database comprising a cohort of thirty-one soldiers who were healthy pre-deployment, deployed to Iraq or Afghanistan for at least one year, then developed new-onset respiratory symptoms during and post-deployment. All the soldiers underwent extensive evaluation of occupational and environmental exposures. Clinical indices included: (1) asthma control test; (2) exhaled nitric oxide concentration; (3) spirometry; (4) body box plethysmography with diffusion capacity; (5) impulse oscillometry; (6) chest CT-scan; (7) lung biopsies when available; and (8) respiratory muscle strength. We also reviewed soldiers’ demographics, including age, sex, BMI, and smoking status. Twenty-one subjects completed non-invasive pulmonary diagnostic testing for maximum expiratory pressure (MEP), a measure of respiratory muscle strength, and 22 underwent airway reactance (X5Hz) which can diagnose airway hyperresponsiveness, and (D5–D20%) which indicates distal airways narrowing. Five military personnel who previously underwent biopsies were referred under the guidance of Northwell IRB: 17-0140-FIMR Feinstein Institution for Medical Research “Clinicopathologic characteristics of Iraq Afghanistan War Lung Injury.” to conduct a minimal-risk, retrospective investigation of human subjects.

### Pulmonary-function and impulse oscillometry testing

We measured results of spirometry, lung volumes, and diffusing capacities according to the guidelines of the American Thoracic Society, interpreted from the findings using the standards of Crapo and colleagues^23–25^. For impulse oscillometry, we followed the guidelines put forth by the European Respiratory Journal and the manufacturer’s guidelines (Jaeger, Vyntus IOS)^26,27^.

### High-resolution computed tomography

Of the thirty-one soldiers in our cohort, eighteen underwent helical computed tomography (CT) scanning without the use of intravenous contrast material. Sagittal and coronal images were reconstructed into 3 mm, contiguous, standard algorithmic images and high-resolution images with a slice thickness of 1 mm every 10 mm. Studies were performed using automatic exposure control (radiation dose reduction software) to minimize patient doses while obtaining diagnostic image quality scans. The administered radiation doses were 5.404 mSv. Transaxial high-resolution images with a thickness of 1.25 mm at 10 mm increments were obtained with inspiration and expiration, and in the supine and prone positions.

### Pathological analysis of lung tissue

Two board-certified pathologists (Michael Esposito, MD; Stephen McClain, MD) examined the lung-biopsy samples in a blinded fashion. The slides were stained with hematoxylin and eosin or Movat's pentachrome for connective-tissue staining. Slides were checked for the presence of carbonaceous particles. Masson’s trichrome connective-tissue staining and Sirius red were also used in many of the specimens. Some had Prussian blue staining for iron. All samples were checked with polarized light for polarizable crystals/particulate matter/dust. These slides were also examined with darkfield microscopy using the microscope’s built-in polarizer and analyzer filters.

The case definition for constrictive bronchiolitis was the presence of extrinsic narrowing of the luminal wall caused by subepithelial fibrosis, smooth-muscle hypertrophy in membranous bronchioles (non-cartilaginous airways having a complete fibromuscular wall), or both in a patient with otherwise normal lung parenchyma. Constrictive bronchiolitis was defined as an increase in wall thickness of more than 20%, as compared with normal thickness.

In addition to the pathologists’ analysis, our team collaborated with a physicist and a geochemist. Each membranous bronchiole lung tissue was examined for polycyclic aromatic hydrocarbons (Raman spectroscopy, Stony Brook University Geosciences), metals, titanium connected to iron, and for oxidized metals, (Brookhaven National Laboratory, National Synchrotron Light Source II Beamline 5-ID), inflammatory cell accumulation and fibrosis, neutrophil extracellular traps (Cold Spring Harbor Laboratory Cancer Biology Laboratory). We analyzed the Ramen curves for polycyclic aromatic hydrocarbons (PAH) and attempted to match our data to that of JP-8. We chose PAH because they are a class of chemicals that occur naturally in fuel. They result from burning fuel. PAHs can bind to or form small particles in the air and can be inhaled. PAHs can also pass through the skin. The majority of motor vehicles were powered by JP-8 fuel^9^.

### National synchrotron light source II bean 5-ID

All samples were thin sections prepared by A. Szema and his team.

- Samples are biopsies of soldier lungs as well as a healthy lung for reference.
- Biopsy samples were embedded in paraffin, thin sections were taken for these studies.
- Sections were either placed on ultralene foil or on polymeric microscopy slide for support during the measurements.
- Samples have been numbered, making the sections anonymous for scientists at NSLS-II. **Only medical scientists know connection to patients.**

PyXRF and Athena software have been used. Both packages are open source and can be found on github.

http://nsls-ii.github.io/PyXRF/

https://bruceravel.github.io/demeter/

There is no written permission necessary as this is open-source.

PyXRF: Li et al.^28^.

Athena: Ravel et al.^29^

X-ray fluorescence (XRF) mapping and X-ray absorption near-edge fine structure (XANES) spectroscopy has been used at beamline 5-ID, the sub-micron resolution X-ray spectroscopy (SRX) beamline, of NSLS-II^30,31^. Samples of lung biopsies have been prepared as thin sections on polymeric microscopy slides or ultralene foil. These slides have been mounted into the beamline for XRF mapping and XANES spectroscopy. The optical system of beamline 5-ID allows for focusing X-rays to a spot of about 0.5 µm diameter through which the samples were raster-scanned. The interaction of X-rays with the elements within the sample causes XRF radiation which is collected with an energy dispersive silicon drift detector. Thus, a full XRF spectrum for each individual scan point is created. To identify individual elements, the XRF spectra are fitted using the software PyXRF^28^. With this method, maps showing the distribution of elements were created. Using these maps, hotspots of Fe and Ti have been identified for chemical analysis. By setting a hotspot into the X-ray focus of the beamline, subsequently fine-tuning the X-ray energy of the incident beam across the K-absorption edge of the element of interest, a XANES spectrum from this hotspot can be obtained. This technique has been used with Fe and Ti to obtain information about the chemical state of these elements. The spectra have been analyzed using the Athena software^29^.

### Neutrophil extracellular traps

To stain for neutrophils and NETs, paraffin embedded sections of lungs were first de-paraffinized, and re-hydrated using standard protocols. Slides were boiled in Tris–EDTA buffer (pH = 9) for 8 min for antigen retrieval and not quenched for H202 afterward. Sections were then blocked with Fc Receptor blocker (#NB309, Innovex) for 30 min, and incubated with 1X donkey blocking buffer (5% donkey serum, 2.5% BSA, 0.1% Triton X-100 in PBS) for one hour. Mouse anti-human MPO (1:500, MAB3174; R&D Systems) and rabbit anti-human citrullinated histone H3 (1:250, ab5103; Abcam) antibodies were incubated together in 0.5X donkey blocking buffer at 4 C overnight. Then sections were incubated with Alexa Fluor 488 conjugated donkey anti-mouse (1:400, A21202, Thermo Fisher Scientific) and Alexa Fluor 568 conjugated donkey anti-rabbit (1:400, A10042, Thermo Fisher Scientific) secondary antibodies together in 0.5X donkey blocking buffer at room temperature for one hour. 4′,6-diamidino-2-phenylindole (DAPI) was used as a nuclear counterstain (Thermo Fisher Scientific, D1306). Images were collected at 40 × magnification using a Leica TCS SP8 confocal microscope (40 × magnification) and were processed with Leica LAS X software.

### Raman methods

Raman spectroscopy is a chemistry technique to provide a structural fingerprint by which molecules can be identified. Raman spectra were collected from undyed and deparaffinized lung biopsy sections using a WiTEC alpha300R confocal Raman imaging system in the Center for Planetary Exploration (CPEx) at Stony Brook University. The imaging system is coupled to a 532 nm neodymium doped yttrium-aluminum-garnet (Nd:YAG) laser with a spot size of ~ 0.8 µm using a 50 × (0.80 NA) objective lens. The laser power was set to ~ 3 mW at the sample surface to avoid damage to the samples.

For each sample, we acquired numerous point Raman spectra (Figs. 5, 6) using a total integration time of 60 s per spectrum to ensure a high signal to noise ratio from small carbon-bearing regions in the samples. Reflected light images were taken before and after sampling to verify that no thermal damage had occurred as a result of exposure to the Raman laser. We also acquired a Raman map of one sample with 500 nm/pixel spatial sampling and 0.5 s total integration time per spectrum.

All Raman spectra showing high integrated band intensities for the 1350 cm^−1^ and 1575 cm^−1^ carbon D and G bands were analyzed further and the associated spectra were subject to a shape-based baseline correction between 900 and 2000 cm^−1^ using the WiTEC Project software and curve fitting using the Igor Pro (Wavemetrics, Lake Oswego, OR, USA) to determine the band parameters.

### Curve fitting and temperature calculation

To characterize the variability of the carbon D and G bands in the Raman spectra, several parameters can be used to quantitatively describe the collected spectra. These parameters include peak position (ω), full-width-half-maximum (Γ), and intensity (I). Collected raw spectra were fit using the 2 Lorentzian band fitting method. This fitting method was chosen to remain consistent and allow for comparison with previous studies^32–34^. Peak position (w_D_ and w_G_), band intensity (I_D_ and I_G_) as well as the FWHM (Γ_D_ and Γ_G_) of the characteristic D and G bands were then extracted from the fit spectra. The band characteristics can be used to calculate the peak temperatures that the carbon particles were subjected to using the models of Busemann et al.^33^ and Cody et al.^34^:

1 $$PT_{Bus} \left( {^\circ C} \right) = 931 - 5.10\left( {FWHM_{D} } \right) + 0.0091(FWHM_{D} )^{2}$$

2 $$PT_{Cody} \left( {^\circ C} \right) = 899.9 - 3\left( {FWHM_{D} } \right) + 0.0014(FWHM_{D} )^{2}$$

### Statistical analysis

Characteristics of the patients were tabulated and the means and standard deviations of pulmonary function testing and impulse oscillometry parameters were calculated. These means were compared to those derived from sixty-nine healthy asymptomatic active service military control subjects in a previously published report using Welch’s t-test^35^.

### Ethics approval

All methods were carried out in accordance with relevant guidelines and regulations. All experimental protocols were approved by Feinstein Institute for Medical Research IRB institutional Review Board Northwell IRB: 17-0140-FIMR Feinstein Institution for Medical Research “Clinicopathologic characteristics of Iraq Afghanistan War Lung Injury”.

### Informed consent

Informed consent was obtained from all subjects and/or their legal guardian(s).

## Results

### Patients

Among the 31 subjects (26 male, 5 female), the median BMI was 29.9 ± 5.2 and age was 36 ± 9.4 (range, 30 to 65). (Table 1) In addition, 25 were lifetime nonsmokers (81%), zero were active smokers, and 6 were former smokers (19%, mean pack-years 10.55 ± 6 pack-years). None of the subjects had a history of asthma pre-deployment.

**Table 1:** Demographic, military location and exposure, radiologic, and pulmonary data from 31 veterans deployed to the Middle East between 2003 and 2015.

Variable	Value (n)	Percentage ((n/31) × 100%) (%)
**Age**		
30–39 years old	20	61
40–49 years old	6	24
50–59 years old	3	9
60–69 years old	2	6
**BMI†**		
Data absent	4	13
Underweight (< 18.5)	0	0
Normal weight (18.5–24.9)	3	10
Overweight (25–29.9)	8	26
Obesity Class 1 (30–34.9)	12	39
Obesity Class 2 (35–39.9)	3	10
Extreme Obesity Class 3 (> 40)	1	3
**Smoking status**		
Current	0	0
Former	6	19
Never	25	81
Deployment location(s)*		
Iraq	23	74
Afghanistan	9	29
Other countries	5	16
**Wartime exposure(s)∆**		
Burn Pit	30	97
Sandstorm	16	52
IED	13	42
**Computed tomography (multiple findings were seen per individual soldier)**		
Data absent	13	42
Normal	5	16
Bronchiectasis	1	3
Atelectasis	8	26
Expiratory ground glass	2	6
Expiratory mosaic pattern	3	10
Air trapping	4	13
Pleural thickening	3	10
Lower lobe consolidative opacities	1	3
**Pulmonary function testing**		
Data absent	2	6
Normal	26	84
Obstructive lung disease	0	0
Restrictive lung disease	3	10
**Respiratory muscle strength (Maximal expiratory pressure or MEP)**		
Data absent	10	32
Normal respiratory muscle strength	0	0
Reduction in respiratory muscle strength	21	68
**Exhaled nitric oxide (FeNO)**		
Data absent	12	39
Normal FeNO	18	58
Abnormal FeNO	1	3
**Carbon monoxide diffusing capacity**		
Data absent	9	29
Normal DLCO	21	68
Abnormal DLCO	1	3
**Impulse oscillometry**		
Data absent	9	29
Normal airways reactance	1	3
Airways hyperresponsiveness (X5)	21	68
Normal airway resistance	12	39
Distal airway narrowing (D5–20)	10	32

^†^BMI categories^35^.

*Some soldiers were deployed to more than one location.

30 out of 31 soldiers in our study had a minimum deployment duration of one year with exposure to burn pits on a daily basis, 24 h a day. From 2003 to 2015, the mean deployment duration was 2.4 years. 23 soldiers in our review were deployed to Iraq, 9 to Afghanistan, and 5 to other locations: Djibouti, Saudia Arabia, UAE, Kuwait, and Qatar. These countries had documented burn pits^36^. One of the thirty-one soldiers did not report exposure to burn pits but was exposed to sandstorms while stationed in Iraq. Nine deployed soldiers independently indicated they were either downwind or at least one hundred yards away from burn pits, often located at the perimeter of their base fields, reporting the distinct smell of the fumes. One cited personal exposure to black plumes.

Furthermore, while all 31 soldiers were exposed to varying concentrations of ambient dust in the air, 16 reported exposures to one or more dust storms characterized as periods of high winds blowing dust and/or sand into the air often disrupting lines of sight. Of these 16 soldiers: one was exposed to just sandstorms, seven to sandstorms and burn pits, and eight to sandstorms, burn pits, and IED detonations. 13 were exposed to detonated IEDs; of which, five to both burn pits and IEDs and eight to sandstorms, burn pits, and IEDs.

Before their exposure to these airborne hazards, all thirty-one soldiers had met the Army Physical Fitness Test requirements, two were triathletes, and none complained of respiratory issues before deployment. Soldiers were examined and deemed fit for deployment at Fort Hood, Texas. Soldiers developed exertional dyspnea and chest tightness during and following deployment, many citing a drop in their two-mile run times. However, soldiers were seen by our practice on average, 8 years following their return home.

Of the thirty-one patients, twenty-nine were examined by our team, while two had their charts and lung biopsies forwarded by external medical facilities.

We focused on patients who underwent non-invasive pulmonary tests, maximum expiratory pressure (MEP), lower airway reactance (X5Hz), and peripheral/distal airway resistance (D5-D20%), because of their mean divergence from percent predicted values. We studied these test results in relation to an anonymous control cohort of sixty-nine previously deployed soldiers as well as to the manufacturer's civilian control cohort (Table 2). Five soldiers had their lungs biopsied. Control comparison biopsies were from the Stony Brook University bank of five histologically normal civilians.

**Table 2:** Pulmonary function, and impulse oscillometry testing in the 31 soldier cohort.

Variable	Normal percent predicted (%)	Military control subjects	Soldiers	*P*-value (Welch's T-Test)	Between-group difference (95% CI)
**Age (yr)**	N/A	27.3 ± 7.5	40.4 ± 9.4	< 0.001	13.1
**Body mass index**	N/A	25.7 ± 3.3	29.9 ± 5.2	< 0.001	4.2
**Pulmonary function testing**					
FVC (% predicted)	90 ± 10	101.6 ± 10.7	91.4 ± 15.9	0.003	− 10.2
FEV1 (% predicted)	90 ± 10	99.1 ± 9.2	93.9 ± 17.7	0.143	− 5.2
FEV1/FVC	90 ± 10	N/A	81.1 ± 5.3	N/A	N/A
FEV1/FVC (% predicted)	90 ± 10	97.4 ± 5.0	101.3 ± 7.5	0.014	3.9
TLC (% predicted)	90 ± 10	99.6 ± 12.0	92.8 ± 14.7	0.053	− 6.8
DLCO (% predicted)	90 ± 10	90.6 ± 12.6	100.6 ± 15.3	0.009	10.0
MVV (% predicted)	90 ± 10	96.2 ± 18.0	92.2 ± 23.7	0.473	− 4.0
**Inspiratory/Expiratory pressures**					
MIP (cm H_2_O)	90 ± 10	108.9 ± 32.1	81.5 ± 27.2	< 0.001	− 27.4
MIP (% Predicted)	90 ± 10	N/A	69.2 ± 26.1	N/A	N/A
MEP (cm H_2_O)	90 ± 10	93.5 ± 34.7	90.0 ± 26.4	0.625	− 3.5
MEP (% Predicted)	90 ± 10	N/A	41.2 ± 10.5	N/A	N/A
**Impulse oscillometry**					
X5Hz (% Predicted)	90 ± 10	N/A	− 1367.9 ± 1584.2	N/A	N/A
D5–D20%	90 ± 10	N/A	23.4 ± 16.9	N/A	N/A

Military control subjects^35^.

### Pulmonary-function and impulse oscillometry testing

Of the thirty-one soldiers, twenty-nine underwent pulmonary function testing. Twenty-six showed normal spirometry and three exhibited a restrictive ventilatory defect. Nineteen underwent carbon monoxide diffusion capacity and exhaled nitric oxide tests: the results for eighteen were normal, while one revealed below normal diffusion capacity, and a separate individual displayed elevated exhaled nitric oxide levels (abnormal).

Impulse oscillometry demonstrated twenty-one out of 22 soldiers had abnormally elevated reactance levels at 5 Hz (X5Hz); 10 out of 22 also had abnormally elevated differences in resistances between 5 and 20 Hz (D5–20%). Patients exposed to all three airborne hazards (sandstorms, burn pits, and detonated IEDs) during deployment had an average X5Hz of − 1789.7% predicted, while those exposed to sandstorms and burn pits averaged at − 1358.2% predicted, followed by − 1223.9% predicted for those only exposed to burn pits. All twenty-one patients who underwent maximum expiratory pressure (MEP) were below 80% of maximal predicted values.

Additional analyses for Table 2:

**Table:** 

Variable	n	N (%) Abnormal*
**Pulmonary function testing**		
FVC (% predicted)	29	5 (17.2%)
FEV1 (% predicted)	29	6 (20.7%)
FEV1/FVC	29	0 (0%)
FEV1/FVC (% predicted)	29	0 (0%)
TLC (% predicted)	23	3 (13.0%)
DLCO (% predicted)	22	1 (4.6%)
MVV (% predicted)	22	6 (27.3%)
**Inspiratory/Expiratory pressures**		
MIP (% Predicted)	21	14 (66.7%)
MEP (% Predicted)	21	21 (100%)
**Impulse oscillometry**		
X5Hz (% Predicted)	22	21 (95.5%)
D5-D20%	22	10 (47.6%)

*Percentages calculated out of those with non-missing data. Abnormal defined as: X5Hz, > 250% predicted; all other parameters, < 80% predicted, except D5–20 > 20%.

### Lung-biopsy findings

**Figure 1:**
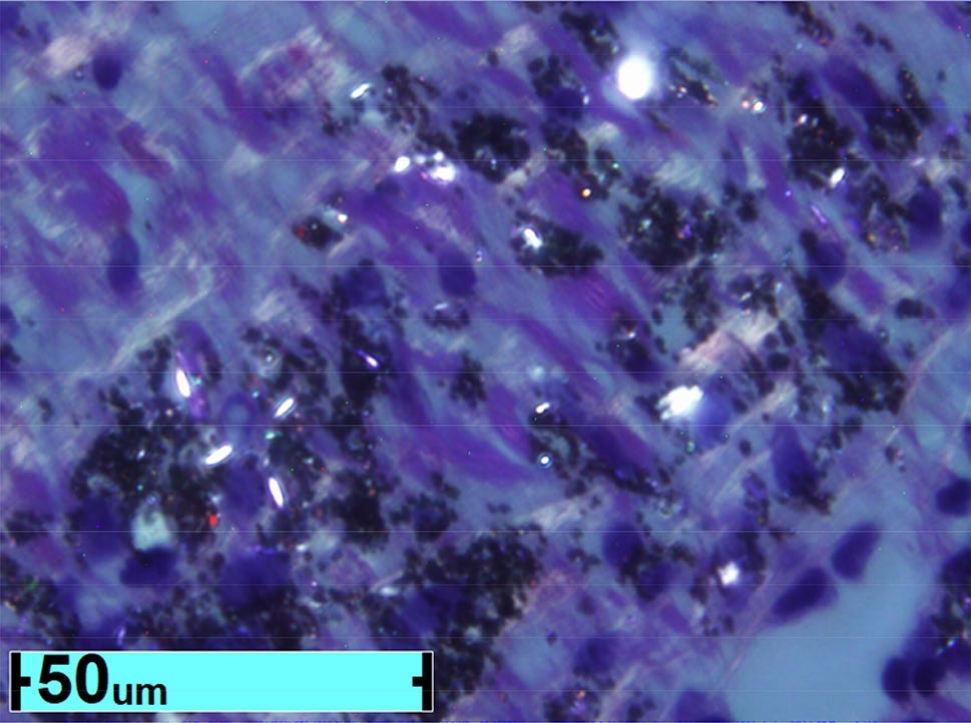
Representative lung biopsy from soldier exposed to burn pits has black carbonaceous particles and white refractile dust crystals amidst fibrosis. These black and white particles have polycyclic aromatic hydorcarbons and contain oxidized metals titanium and iorn.

**Figure 2:**
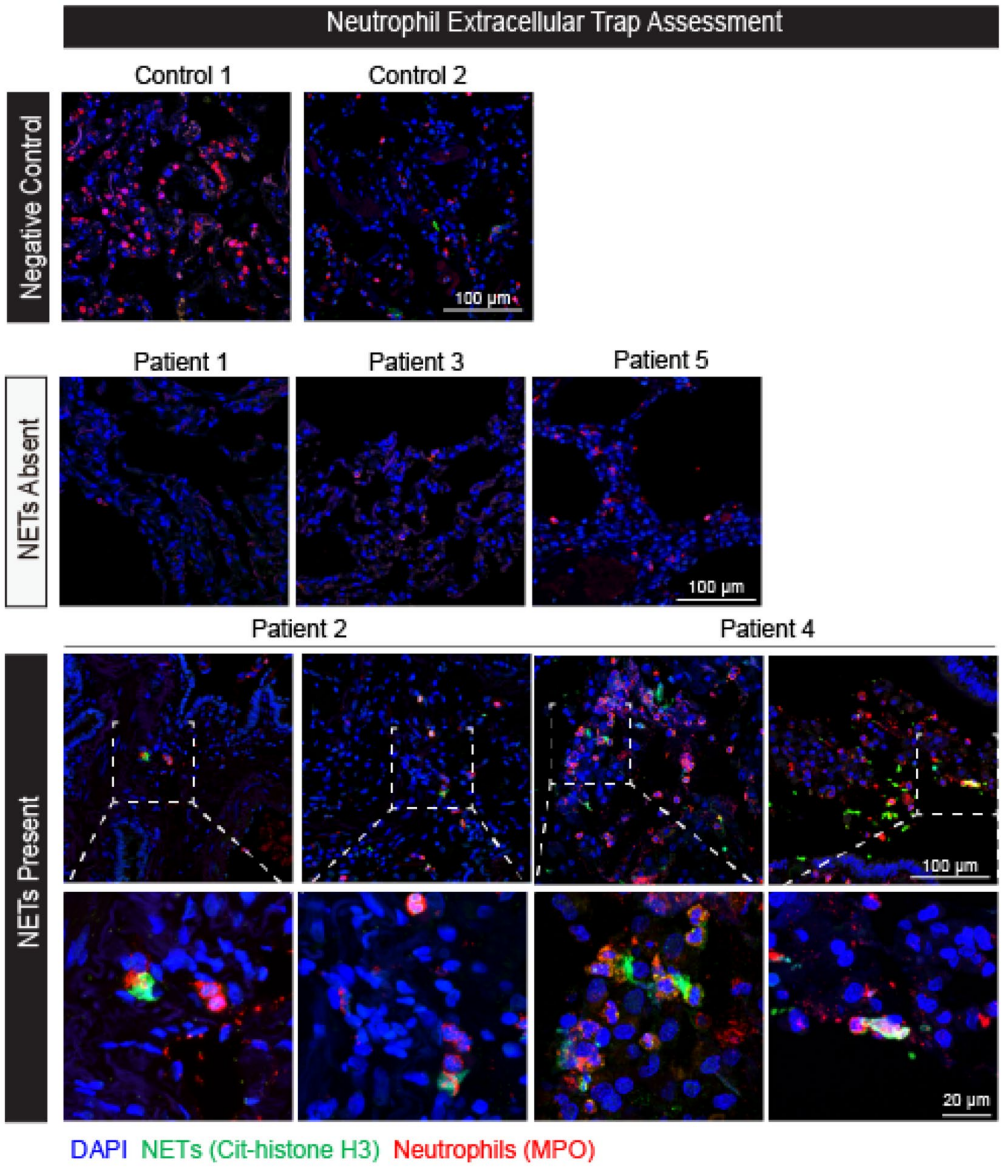
Representative confocal microscopy of lung parenchyma for Neutrophil Extracellular Traps (NETs) staining from the patient cohort with non-fibrosed controls, patients without NETs, and patients with NETs. NETs were indicated by immunofluorescent staining for myeloperoxidase (red), citrullinated histone H3 (green), and DAPI (blue). Top row (Negative Control): Imaging from the lung parenchyma from non-fibrosed controls; Scale bar, 100 μm. Middle row (NETs Absent) : Imaging from the lung parenchyma of patients 1, 3, and 5 with NETs absent; Scale bar, 100 μm. Bottom row (NETs Present): Higher-magnification images of the boxed areas of Patient 2 and 4 are shown at bottom row; scale bar, 20 μm.

A diagnosis of constrictive bronchiolitis was made after reviewing the five biopsies taken within our cohort of thirty-one soldiers. (Table 3) A representative biopsy is shown in Fig. 1. Neutrophil Extracellular Trap staining is described in Fig. 2.

**Table 3:** Biopsy results for five soldiers deployed to the middle east between 2003 and 2015.

Variables	Patient 1 (Female DE)	Patient 2 (Male FL)	Patient 3 (Male PA)	Patient 4 (Male WV)	Patient 5 (Female MI)
Atelectasis	1	1	1	1	0
Alveolar Wall Destruction (COPD)	2	3	2	2	3
Fibrosis (Ashcroft score score 1–6 with 6 severe)	6	6	2	6	1
SiriusRed collagen (0 to + + +)	3	3	1	3	1
Elastic fibers (EVG score)	3	1	0	2	0
**Inflammatory Infiltrate**					
PMN	Few	Few	2 score	Few	Few
Lymphocytes per high powered field	0	0	Few-moderate	0	0
EOS per high powered field	few	0	0	0	0
**Pulmonary Ossification number of areas**	2	1	0	1	0
**Bronchiectasis**	0	0	0	0	0
PAH	Present	Present	Present	Present	Present
Carbonaceous particulate matter (grade 0 to 4)	3	3	4	3	1
Refractile or polarizable crystals per high powered field	2	1	4	3	1
Iron + Macrophages	3	3	0	1	1
Titanium (present)	Yes	Yes	Yes	Yes	Yes
Iron in lung tissue (present)	Yes	Yes	Yes	Yes	Yes
Oxidized metal (present)	Yes	Yes	Yes	Yes	Yes
Combustion temperature	245-246C Buseman 298–303 Cody	218-260C Buseman 194–328 Cody	249-416C Buseman 307-528C Cody	229-560C Buseman 252-653C Cody	269-313C Buseman 346-412C Cody
Neutrophil extracellular traps (NETS)	No	Yes	No	Yes	No

### Imaging and spectroscopy studies

Of the thirty-one soldiers, we had chest CT scans for 18 (58%). Of those 18, 13 or 72% of soldiers who received a CT scan presented some indication of abnormality; one had bronchiectasis, eight had atelectasis, two had expiratory ground glass, three had an expiratory mosaic pattern(s), four had air trapping, three had pleural thickening, and one had lower lobe consolidative opacities.

The XRF maps obtained at beamline 5-ID show the distribution of metals and other elements in the thin sections. In Fig. 5, left, a part of the thin section area of sample is displayed using the visible light microscope of beamline 5-ID. This area has been raster-scanned to obtain the XRF-map shown on the right. The incident X-ray energy has been set to E = 12 keV to enable detecting transition metals and biologically relevant elements such as zinc. Using a step size of 1.5 µm, an area of 600 × 750 microns with 400 × 500 pixels has been scanned. The dwell time per pixel has been 0.1 s, i.e. the acquisition of the picture took approx. 13 h. For the XRF map on the right (Fig. 4), an RGB overlay of the image area has been created showing the distribution of iron (red) and Ti (green) in front of a sulfur (blue) background. It appears that almost all Ti particles are co-localized with Fe, which is the reason for them appearing yellow. Only few Ti particles can be identified by their green color as being separate.

**Figure 3:**
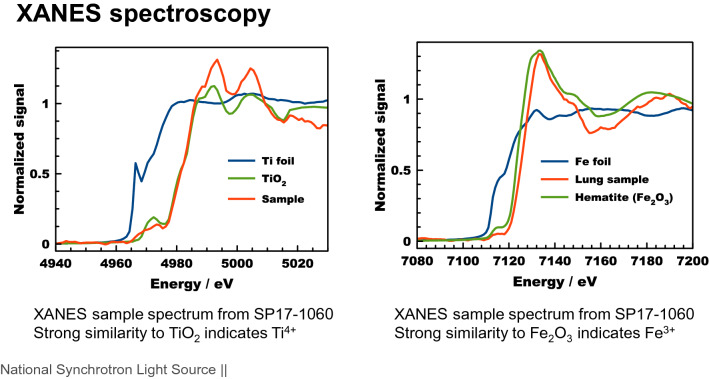
XANES spectra, taken beamline 5-ID of NSLS-II. Left: XANES spectra of an Fe-foil, a hematite and the lung sample at the Fe K-edge. Right: XANES spectra of an Ti-foil, TiO_2_ , and the lung sample at the Ti K-edge.

**Figure 4:**
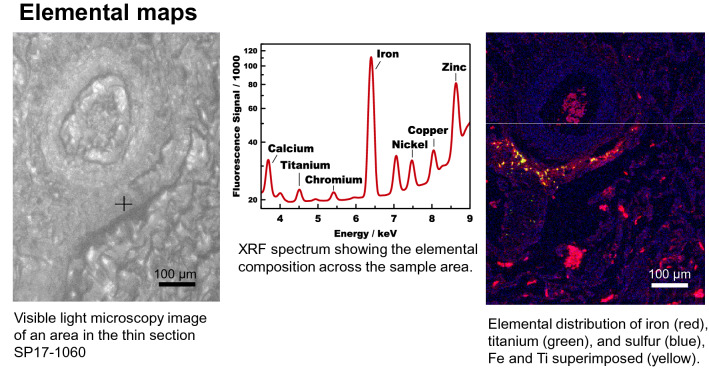
Lung sample. Left: Visible light microscope image of the thin section area investigated using XRF mapping. Right: XRF map of the same area showing the distribution of iron (red) and Ti (green) in front of a sulfur (blue) background. Many Ti particles appear yellow due to the co-localization with Fe.

Applying XANES spectroscopy to the hotspots of the sample showing Ti and Fe, information about the chemical state of these elements has been obtained. Figure 4 shows XANES spectra that have been measured in the area marked with an orange square in Fig. 3. In addition to spectra from the sample, spectra from reference materials have been measured. Figure 4 left shows spectra obtained from a pure iron foil (blue), from the mineral hematite (Fe_2_O_3_, green), and from the sample (red). Generally, and true for all absorption spectra of elements, the steep rise in absorption shifts to higher energies with a higher oxidation state of the element. The shape of the sample spectrum matches quite well the shape of the hematite spectrum, which indicates first that the oxidation state of Fe is 3+ and second that Fe present here is most likely an iron oxide very similar to the reference mineral. Figure 4 right shows spectra obtained from a pure Ti foil (blue), from TiO_2_ (green), and from the sample (red). Using the same rationale, it can be stated that Ti is found in a 4+ oxidation state, most likely as a Ti oxide.

Synchrotron Summary:

- Lung biopsy samples have been investigated in three experiment runs at the SRX beamline, 2018, 2019, and 2021.
- Elemental maps using XRF were taken from the thin sections.
- *Metal particles have been found in the few-µm size range* and the elemental composition of these particles has been mapped.
- Among other metals, iron and titanium have been identified in these particles.
- XANES spectra were taken from the particles to obtain information about the chemical speciation of Fe and Ti.
- *All metals in all samples have been found in an oxidized state*,
- such as Fe^3^ + or Ti^4+^.

**Figure 5:**
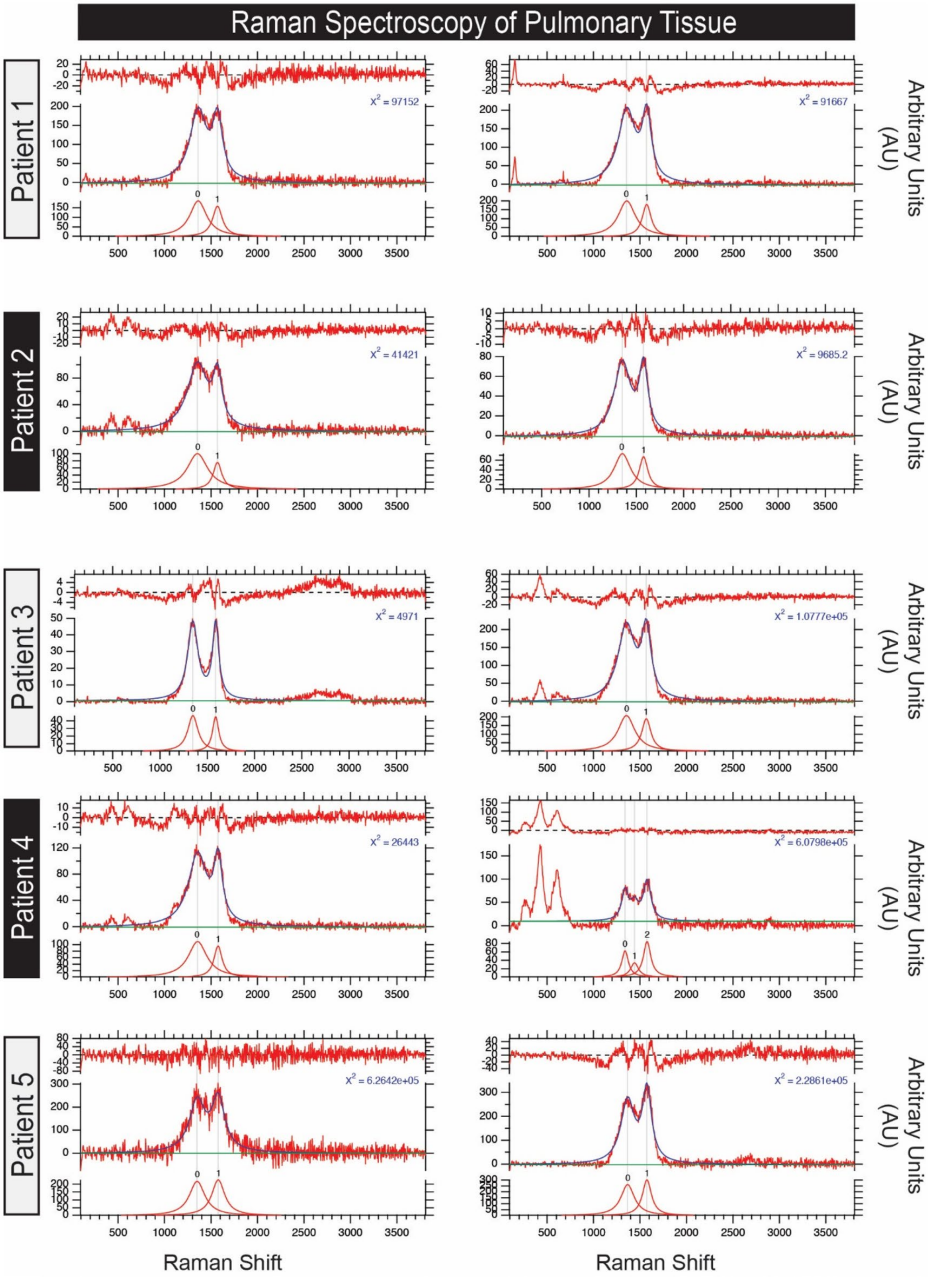
Raman shift plotted against signal intensity for each patient. Tissue from five cohort patients display Raman spectra consistent with polycyclic aromatic hydrocarbons (PAH). Two readings of carbonaceous debris per patient are shown. The carbon D band (~ 1350 cm^−1^) is utilized to estimate the peak combustion temperatures according to the models of Busemann et al.^33^ and Cody et al.^34^. Each plot shows the spectrum and modeled fit (center), the Lorentzian curves used to model the carbon D and G bands (bottom), and the model residual (top).

**Figure 6:**
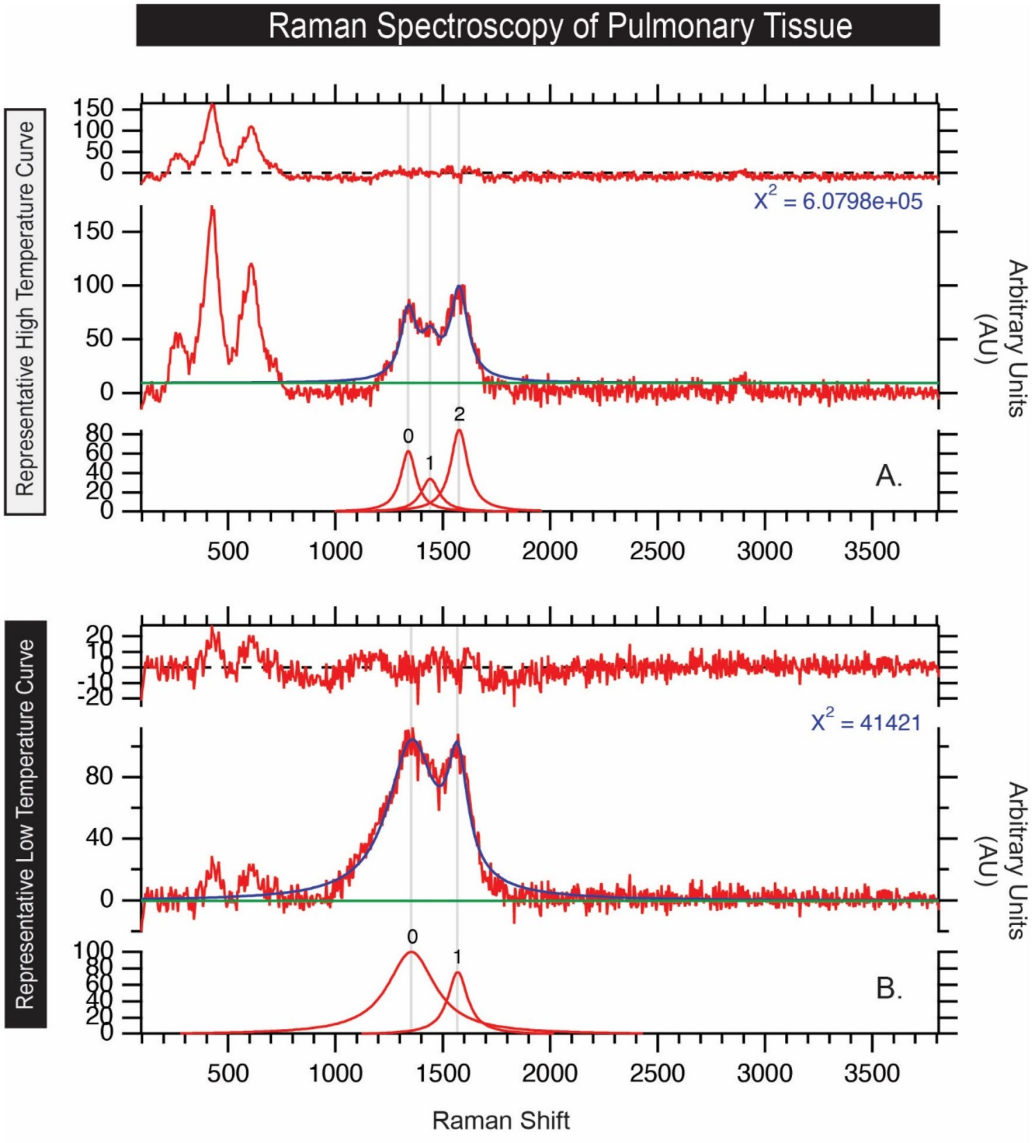
Raman shift plotted against signal intensity. The representative polycyclic aromatic hydrocarbon (PAH) curves are associated with the highest and lowest temperatures calculated using the Busemann et al.^33^ model, in A and B, respectively.

Figure 5 shows Raman spectroscopy of pulmonary tissue indicates polycyclic aromatic hydrocarbons in all biopsies. As a representative example, Fig. 6 calculates highest and lowest temperatures of carbonaceous particles/polycylic aromatic hydrocarbons in the lung biopsy.

## Discussion

We show in this manuscript a direct link between veteran’s lung injury and burn pits exposures. Using sophisticated techniques such as the National Synchrotron Light Source II and Ramen Spectroscopy, respectively, our team has detected burned products of incomplete combustion, with anthropogenic, burned oxidized metals in fixed mathematical ratios not found in nature for the former, and polycyclic aromatic hydrocarbons fitting the pattern of JP-8 and clearly representative of petrol with peak combustion temperatures of 300* °C.* Other supportive data linking veterans' lung injury to burn pit exposures in our cohort include the finding of carbonaceous particles within the burned particulate matter. We show all of these injuries are deleterious because uniformly there is fibrosis and inflammation in both soldiers’ biopsies and our animal model. In particular, all soldiers with biopsies are symptomatic of new-onset dyspnea. These findings duplicate our previously published mouse model of burn pit base dust exposure which also shows these metals are reflected in polarizable crystals. In addition, our review of representative biopsies by Dr. Robert Miller's cohort at Vanderbilt shows identical pathology.

In this study, we have identified a cohort of previously healthy soldiers who experienced new-onset respiratory symptoms beginning in military theater, predominately in Iraq and Afghanistan, and persisting post-deployment. We can associate their lung injury with continuous exposure over an average of 2.4 years to airborne hazards, including dust storms (30 soldiers), burn pits (16 soldiers), and IEDs (13 soldiers).

Their clinical endotype shows a reduction in respiratory muscle strength (Maximum Expiratory Pressure or MEP), which may predict neuromuscular dysfunction. Note that BMI may be misleading in muscular athletes. Concomitantly, they frequently exhibit airways hyperresponsiveness and distal airways narrowing. Lung tissue demonstrates dust particles, which are polarizable and like that reported by Miller, and colleagues^5^**.** Biopsies are reflective of our mouse Iraq and Afghanistan inhalation models. Also seen are carbonaceous particles (less than 5 µm), polycyclic aromatic hydrocarbons (PAHs), metals (titanium bound to iron in fixed mathematical ratios of 1:7, supporting an anthropogenic source), constrictive bronchiolitis, and parenchymal fibrosis.

Massive daily inhalation of n-hexane is a plausible byproduct of burning plastic water bottles and Styrofoam breakfast trays. Several studies have indicated a potential correlation between n-hexane exposure and the subsequent development of small fiber neuropathy in human subjects^37,38^. Inhalational lung injury explains airway dysfunction. Detection of polycyclic aromatic hydrocarbons and carbonaceous particles in lung tissue with oxidized metals strongly suggests that burn pit plume exposure was the etiologic mechanism. Oxidation of metals supports the concept that the metals were burned.

In the 2019 Respiratory Health after Military Service in Southwest Asia and Afghanistan. An Official American Thoracic Society Workshop Report, colleagues note that dust respiratory illness prior to the 2003 Iraq war led to increased mortality, cardiovascular mortality, pneumonia, asthma, rhinitis, silicosis, bronchitis, and emergency room visits. However, we are the first to report this new Iraq-Afghanistan War Lung Injury (IAW-LI), with a unique burned histopathology. A PubMed search on June 22, 2022, using the terms “Iraq; dust storms; respiratory” was unable to find articles prior to 2003 linking dust storms to respiratory illness among Iraq citizens, including children. Comparing the region’s pre-wartime concentration of particulate matter in ambient dust storms with post-2003 concentration and consequential respiratory effects from exposure is difficult due to a lack of prior large-scale epidemiologic studies^39^. Recent studies, however, estimate annual PM_2.5_ average concentrations in many U.S. bases and large cities (e.g., Bagdad, Balad, Kuwait city, Karbala, Najaf, and Diwaniya) to be above 45 μg/m3 with weekly averages as high as 150 μg/m^3^, peaking in 2008, all of which are greater than current U.S. and WHO standards of 10 μg/m^3^^39,40^.

Our methodology emphasizes that quaternary care and sophisticated analyses may be needed when spirometry and CT scans are insufficiently sensitive to detect lung disease. While the diagnosis of Iraq Afghanistan War lung injury (IAW-LI) may only be definitively established by tissue analysis, we show that non-invasive methods correlate with respiratory muscle weakness, distal airways hyperresponsiveness/narrowing, and constrictive bronchiolitis in exposed warfighters.

## Data Availability

The datasets used and/or analyzed during the current study are available from the corresponding author upon reasonable request.
