# Author Correction: Iraq/Afghanistan war lung injury reflects burn pits exposure

**DOI:** 10.1038/s41598-022-26538-8

**Published:** 2022-12-22

**Authors:** Timothy Olsen, Dennis Caruana, Keely Cheslack-Postava, Austin Szema, Juergen Thieme, Andrew Kiss, Malvika Singh, Gregory Smith, Steven McClain, Timothy Glotch, Michael Esposito, Robert Promisloff, David Ng, Xueyan He, Mikala Egeblad, Richard Kew, Anthony Szema

**Affiliations:** 1 University of Rochester School of Medicine and Dentistry, Simon Business School, University of Rochester, Rochester, USA; 2 Yale University School of Medicine, New Haven, USA; 3 Columbia University Global Psychiatric Epidemiology Group, NYSPI Columbia University Department of Psychiatry, New York, USA; 4 Northeastern University College of Art, Media, and Design (CAMD) Game Design Program, Boston, USA; 5 Brookhaven National Laboratory National Synchrotron Light Source II Beam ID-5, Upton, USA; 6 Science Coordinator Imaging and Microscopy Program and Department of Geosciences, Stony Brook University, Stony Brook, USA; 7 Brookhaven National Laboratory National Synchrotron Radiation Light Source II Bean ID-5, Upton, USA; 8 Department of Pharmacological Sciences, Stony Brook University, Stony Brook, USA; 9 McClain Laboratories, Smithtown, USA; 10 Center for Space Exploration (CEx) Department of Geosciences, Stony Brook University, Stony Brook, USA; 11 Department of Pathology North Shore University Hospital Northwell Health, Donald and Barbara Zucker School of Medicine at Hofstra/Northwell, Hempstead, USA; 12 Drexel University College of Medicine, Philadelphia, USA; 13 Rockefeller University Department of Cancer Biology, New York, USA; 14 Cold Spring Harbor Laboratory Department of Cancer Biology, Cold Spring Harbor, New York, USA; 15 Department of Pathology Stony Brook University, Stony Brook, NY USA; 16 Division of Pulmonary and Critical Care, Division of Allergy/Immunology, Northwell Health, New Hyde Park, USA; 17 Department of Occupational Medicine, Epidemiology and Prevention, International Center of Excellence in Deployment Health and Medical Geosciences, Donald and Barbara Zucker School of Medicine at Hofstra/Northwell, Hempstead, USA

Correction to: *Scientific Reports* https://doi.org/10.1038/s41598-022-18252-2, published online 29 August 2022

The original version of this Article contained an error in the Abstract.

“Among the five soldiers biopsied, all had constrictive bronchiolitis.”

now reads:

“Among the five soldiers biopsied, all had constrictive bronchiolitis or bronchiolitis or severe pulmonary fibrosis.”

The original Article has been corrected.

